# Sirolimus-coated balloon in all-comer population of coronary artery disease patients: the EASTBOURNE DIABETES prospective registry

**DOI:** 10.1186/s12933-024-02139-9

**Published:** 2024-02-03

**Authors:** Gianluca Caiazzo, Angelo Oliva, Luca Testa, Tay M. Heang, Chuey Y. Lee, Diego Milazzo, Giulio Stefanini, Nicola Pesenti, Antonio Mangieri, Antonio Colombo, Bernardo Cortese, on behalf of the EASTBOURNE investigators

**Affiliations:** 1 U.O.C. UTIC-Cardiologia, P.O. San Giuseppe Moscati - Aversa – ASL Caserta, Aversa, Italy; 2 Department of Biomedical Sciences, Humanitas University, Pieve Emanuele-Milan, Italy; 3 Humanitas Research Hospital IRCCS, Rozzano - Milan, Italy; 4 IRCCS Policlinico San Donato, Milano, Italy; 5 Pantai Hospital Ayer Keroh, Melaka, Malaysia; 6 Sultanah Aminah Hospital Johor Bahru, Johor bahru, Malaysia; 7 ASP S. Giovanni di Dio, Agrigento, Italy; 8 Department of Statistics and Quantitative Methods, Division of Biostatistics, Epidemiology, and Public Health, University of Milano-Bicocca, Milano, Italy; 9 We 4 Clinical Research, Milano, Italy; 10 Cardiovascular Research Group, Fondazione Ricerca e Innovazione Cardiovascolare, Via Vico, 2, Milano, Italy; 11 DCB Academy, Milano, Italy

**Keywords:** Coronary artery disease, Diabetes mellitus, Drug-coated balloon

## Abstract

**Background:**

The outcomes of percutaneous coronary intervention (PCI) in diabetic patients are still suboptimal, and it is unclear if diabetic patients might derive a benefit from the use of drug-coated balloons.

**Aims:**

To evaluate the impact of diabetes mellitus on the outcomes of patients undergoing PCI with sirolimus-coated balloon (SCB) MagicTouch (Concept Medical, India).

**Methods:**

We conducted a subgroup analysis of the prospective, multicenter, investigator-initiated EASTBOURNE registry, evaluating the performance of MagicTouch SCB in patients with and without diabetes. The study primary endpoint was target lesion revascularization (TLR) at 12-month follow-up. Secondary clinical endpoints were major adverse clinical events (MACE), death, myocardial infarction (MI), and BARC 2–5 bleedings.

**Results:**

Among 2,083 enrolled patients, a total of 864 suffered from diabetes (41.5%). Patients with diabetes had a numerically higher occurrence of TLR (6.5% vs. 4.7% HR 1.38, 95%CI 0.91–2.08), all-cause death (3.8% vs. 2.6%, HR 1.81, 95%CI 0.95–3.46), and MACE (12.2% vs. 8.9%; HR 1.26 95%CI 0.92–1.74). The incidence of spontaneous MI was significantly higher among diabetic patients (3.4% vs. 1.5%, HR 2.15 95%CI 1.09–4.25); bleeding events did not significantly differ. The overall incidence of TLR was higher among in-stent restenosis (ISR) as compared to de-novo coronary lesions, irrespectively from diabetes status.

**Conclusions:**

In the EASTBOURNE DIABETES registry, diabetic patients treated with the MagicTouch SCB did not have a significant increase in TLR when compared to non-diabetic patients; moreover, diabetic status did not affect the study device performance in terms of TLR, in both de-novo lesions and ISR.

## Introduction

Despite the significant advances achieved during the last decades in terms of novel devices and tailored therapies, the outcomes of diabetic patients undergoing percutaneous coronary intervention (PCI) for coronary artery disease (CAD) are still poor, since diabetes is per se associated with a greater risk of major adverse cardiovascular events (MACE) and repeated revascularization [1–3]. The introduction of new-generation drug-eluting stents (DES) had a major impact in terms of reduction of device-oriented events, as compared to previous-generation DES; however the benefit is lower in patients suffering from diabetes [4, 5]. Given the higher incidence of restenosis, myocardial infarction (MI), and stent thrombosis after PCI with stent implantation, diabetic patients might derive a benefit from a minimalistic approach based on angioplasty with drug-coated balloons (DCB) without any permanent metallic scaffold implantation – the so-called “leave nothing behind” strategy [6, 7]. DCB are a new, promising innovation in the interventional cardiology field, that may represent a valid alternative to DES. Through a single prolonged inflation, DCB are able to restore an adequate lumen in a stenotic coronary artery and contemporarily transfer an antiproliferative drug from a lipophilic matrix to the vessel wall. Safety and efficacy of DCB have been already shown for the treatment of in-stent restenosis (ISR) and small-vessel de-novo coronary lesions [7–11]. Recently, the advent of the novel MagicTouch (Concept Medical, India) sirolimus-coated balloon (SCB) added another therapeutic option for treatment of coronary artery disease [12]. The all-comer Sirolimus-coated balloon European (EASTBOURNE) registry, is the largest prospective study on DCB so far, evaluating the clinical performance of SCB [13]. In this prespecified sub-analysis, we aimed to evaluate the impact of diabetes mellitus on clinical outcomes among patients treated with SCB and enrolled in the EASTBOURNE registry.

## Methods

### Study design and population

The EASTBOURNE registry (NCT03085823) is a prospective, investigator-initiated, clinical registry that enrolled all-comer patients undergoing PCI with MagicTouch SCB at 38 European and Asiatic centers. The present pre-prespecified analysis evaluated the mid-term efficacy and safety of this SCB in patients with and without diabetes, undergoing PCI for revascularization of both ISR and de-novo coronary lesions.

As previously described [13], to be deemed eligible for the inclusion into the study, patients had to be > 18 years old, presenting with coronary artery disease with clinical indication to PCI, including stable angina, silent ischemia and acute coronary syndrome. Relevant exclusion criteria were the presence of known hypersensitivity or contraindication to aspirin, heparin, P2Y12 inhibitors, sirolimus or contrast media and/or the presence of any of the following lesion characteristics: (1) unsuccessful pre-dilatation of the target lesion (residual stenosis > 50%); (2) severe calcification of the target vessel, (3) highly tortuous culprit vessels; (4) visible thrombus at the lesion site, not treatable with manual aspiration.

### Procedure and device description

The PCI procedure was performed according to current international guidelines and local best practice. Intraprocedural intravenous heparin was administered in order to maintain an activated clotting time higher than 250 s (or > 200 s if glycoprotein IIb/IIIa inhibitors were used, at the operator’s discretion). Aspirin 100–325 mg was given prior to the procedure and a loading dose of ticagrelor 180 mg, prasugrel 60 mg or clopidogrel 600 mg was administered, depending on the clinical presentation of the patient. After the procedure, the antithrombotic regimen was left to the operator’s choice, but a minimum of 30-day dual antiplatelet therapy (DAPT) consisting of aspirin (plus an oral P2Y12 inhibitor was recommended. In case of patients presenting with acute coronary syndrome (ACS) and/or receiving stent implantation, a regimen of 6 up to 12 months of DAPT was advised.

The MagicTouch SCB is a semi-compliant balloon coated with sirolimus encapsulated in a phospholipidic bilayer nanocarrier. Drug nominal dose is 1.27 mg/mm^2^ and the device is available from 10 to 40 mm in length and from 1.50 to 4.00 mm in diameter. The decision whether to use the SCB was left to the operator’s discretion. Lesion preparation was mandatory, and the use of any appropriate device including semi- or non-compliant balloons, atherectomy, scoring balloons, or lithotripsy was allowed. Prolonged inflation of SCB at target lesion for at least 30 up to 60 s was strongly encouraged. The decision to implant a stent after SCB was recommended only in case of acute vessel recoil or flow-limiting residual dissection.

### Data collection, study endpoints and follow-up

Demographic and clinical information were collected through an electronic data system. All clinical events were centrally adjudicated by a blinded committee of physicians who analyzed all documents provided by the centers. The primary endpoint of the study was target lesion revascularization (TLR) at 12-month follow-up. Secondary clinical endpoints were the occurrence of MACE, defined as a composite of cardiac death, acute MI and TLR, the occurrence of each component of MACE, and BARC 2–5 bleedings at 12-month follow-up.

### Statistical analysis

Categorical variables are reported as count and percentage, whereas continuous variables as mean values ± standard deviations (SD) or median and interquartile range (IQR). The t test has been used to assess differences between parametric continuous variables, Mann–Whitney U test for nonparametric variables, the chi-square test or Fisher’s exact test for categorical variables. The overall cumulative risk of TLR was estimated using the Kaplan-Meier method, and the differences among the groups estimated using the log-rank test. Comparisons were evaluated between diabetic and non-diabetic patient groups and further stratified according to the type of target lesion (de-novo coronary lesions vs. in-stent restenosis). The effect of diabetic groups and lesion type on the study endpoints was estimated by a Cox proportional hazards model and expressed as a hazard ratio (HR), 95% CI and p-value. The model was adjusted for potential confounding factors, such as: patient age, hypercolesterolemia, hypertension, multi-vessel disease and predilatation, resulted statistically significant at univariate analysis. To avoid interlesional clustering of the TLR patients who received stents for multiple lesions, Cox regression model of TLR was analyzed per patient. A two-sided P value less than 0.05 was considered statistically significant; all analyses were performed using the R software (R Core Team 2022. A language and environment for statistical computing. R Foundation for Statistical Computing, Vienna, Austria. URL https://www.R-project.org/).

## Results

Between 2016 and 2020 a total of 2,123 patients (2,440 lesions) were enrolled in the EASTBOURNE registry. For the purpose of the present analysis, a total of 2,083 patients (2,162 lesions) with available data were considered. Among enrolled patients, 864 patients (41.5%) had diabetes of whom 32.8% (283 patients) was insulin dependent. In Table 1, baseline and angiographic characteristics of patients are reported stratified according to the diabetic status and type of target lesion (de-novo lesions vs. ISR). Compared to non-diabetic, diabetic patients were older, with a higher incidence of hypercholesterolemia, hypertension, congestive heart failure, presenting more often with a clinical history of previous PCI, myocardial infarction, and multivessel disease. Procedural characteristics are reported in Table 2. No significant differences were found in terms of clinical presentation, number of lesions treated and lesion length; multivessel PCI with study device or stent implantation was performed in a total of 41.9% of patients during index procedure and did not differ between diabetic and non-diabetic patients in the overall population. Notably, albeit patients with de-novo coronary lesions were prevalent (56.3% of the total), among diabetic patients SCB were used more commonly for ISR as compared to de-novo lesions (52.3% vs. 47.7%). Pre-dilatation, requested per protocol, was performed in 91.7% of the cases, more frequently in diabetic patients (94.2% vs. 89.9%, *P* = 0.001); bailout stenting occurred after SCB in 6.9% and 8.3% of the cases in diabetic and non-diabetic patients, respectively (*p* = 0.258). Final angiographic success was achieved in 97.6% of patients, with no differences between diabetic and non-diabetic patients (97.9% vs. 97.3%, *p* = 0.445).

**Table 1:** Baseline clinical characteristics stratified according diabetic status and type of lesion (de-novo lesions vs. ISR)

	Overall population (*N* = 2,083)			Patents with de-novo lesions (*N* = 1,173)			Patients with ISR (*N* = 910)		
	Patients with DM (*N* = 864)	Patients without DM (*N* = 1,219)	p-value	Patients with DM (*N* = 452)	Patients without DM (*N* = 721)	p-value	Patients with DM (*N* = 412)	Patients without DM (*N* = 498)	p-value
Male (%)	691 (80.0)	999 (82.0)	0.281	358 (79.2)	599 (83.1)	0.112	333 (80.8)	400 (80.3)	0.915
Age, mean (SD)	67.21 (10.39)	66.19 (11.84)	0.043	65.08 (11.11)	64.42 (12.21)	0.353	69.53 (8.99)	68.77 (10.79)	0.257
Insulin dependent (%)	283 (32.8)	0 (0.0)	< 0.001	120 (26.5)	0 (0.0)	< 0.001	163 (39.6)	0 (0.0)	< 0.001
Hypercholesterolemia (%)	683 (79.1)	813 (66.7)	< 0.001	345 (76.3)	441 (61.2)	< 0.001	338 (82.0)	372 (74.7)	0.010
Hypertension (%)	711 (82.3)	893 (73.3)	< 0.001	343 (75.9)	497 (68.9)	0.012	368 (89.3)	396 (79.5)	< 0.001
Prior MI (%)	391 (45.3)	503 (41.3)	0.077	152 (33.6)	209 (29.0)	0.107	239 (58.0)	294 (59.0)	0.806
Prior CABG (%)	109 (12.6)	135 (11.1)	0.313	35 (7.7)	46 (6.4)	0.437	74 (18.0)	89 (17.9)	0.999
Prior PCI (%)	605 (70.0)	775 (63.6)	0.003	206 (45.6)	297 (41.2)	0.157	399 (96.8)	478 (96.0)	0.608
Multivessel disease (%)	542 (62.7)	693 (56.8)	0.008	258 (57.1)	373 (51.7)	0.084	284 (68.9)	320 (64.3)	0.157
Heart Failure (%)	90 (10.4)	80 (6.6)	0.002	40 (8.8)	36 (5.0)	0.013	50 (12.1)	44 (8.8)	0.129
LVEF, mean (SD)	50.63 (11.43)	52.51 (10.69)	< 0.001	50.94 (11.88)	53.07 (11.22)	0.003	50.31 (10.93)	51.73 (9.87)	0.042
Creatinine mg/ml, median [IQR]	1.02 [0.86. 1.30]	0.98 [0.82. 1.13]	< 0.001	1.01 [0.84. 1.28]	0.97 [0.82. 1.10]	< 0.001	1.03 [0.87. 1.35]	1.00 [0.81. 1.19]	0.001
Hb g/dl, mean (SD)	13.04 (2.30)	13.63 (1.99)	< 0.001	13.23 (2.28)	13.70 (2.02)	< 0.001	12.84 (2.31)	58.70 (1007.64)	0.356
Clinical presentation									
Stable Angina, (%)	299 (34.6)	407 (33.4)	0.089	152 (33.6)	227 (31.5)	0.260	147 (35.7)	180 (36.1)	0.035
Silent Ischemia (%)	151 (17.5)	258 (21.2)		83 (18.4)	171 (23.7)		68 (16.5)	87 (17.5)	
Unstable Angina (%)	144 (16.7)	220 (18.0)		61 (13.5)	95 (13.2)		83 (20.1)	125 (25.1)	
NSTEMI (%)	207 (24.0)	238 (19.5)		104 (23.0)	153 (21.2)		103 (25.0)	85 (17.1)	
STEMI, < 12 h (%)	38 (4.4)	53 (4.3)		31 (6.9)	36 (5.0)		7 (1.7)	17 (3.4)	
STEMI, > 12 h (%)	25 (2.9)	43 (3.5)		21 (4.6)	39 (5.4)		4 (1.0)	4 (0.8)	

DM: diabetes mellitus; ISR: in-stent restenosis; MI: myocardial infarction; CABG: coronary artery bypass graft; PCI: percutaneous coronary intervention; LVEF: left ventricular efection fraction; Hb: hemoglobin; NSTEMI: non ST-elevated myocardial infarction; STEMI: ST-elevated myocardial infarction

**Table 2:** Periprocedural characteristics stratified according diabetic status and type of lesion (de-novo lesions vs. ISR)

	Overall population (*N* = 2,083)			Patents with de-novo lesions (*N* = 1,173)			Patients with ISR (*N* = 910)		
	Patients with DM (*N* = 864)	Patients without DM (*N* = 1,219)	p-value	Patients with DM (*N* = 452)	Patients without DM (*N* = 721)	p-value	Patients with DM (*N* = 412)	Patients without DM (*N* = 498)	p-value
Multivessel PCI (%)	373 (43.2)	500 (41.0)	0.349	195 (43.1)	328 (45.5)	0.467	178 (43.2)	172 (34.5)	0.009
Number of lesions treated									
1	773 (89.5)	1081 (88.7)	0.223	420 (92.9)	652 (90.4)	0.458	353 (85.7)	429 (86.1)	0.260
2	78 (9.0)	126 (10.3)		30 (6.6)	63 (8.7)		48 (11.7)	63 (12.7)	
3	13 (1.5)	10 (0.8)		2 (0.4)	4 (0.6)		11 (2.7)	6 (1.2)	
4	0 (0.0)	2 (0.2)		0 (0.0)	2 (0.3)		0 (0.0)	0 (0.0)	
Reference vessel diameter, mean (SD)	2.56 (0.68)	2.55 (0.71)	0.748	2.24 (0.50)	2.28 (0.58)	0.200	2.91 (0.68)	2.94 (0.71)	0.554
Lesion length, mean (SD)	18.94 (9.88)	18.55 (8.63)	0.342	19.98 (10.26)	19.05 (9.18)	0.107	17.66 (9.41)	17.68 (7.85)	0.969
Minimal lumen diameter, mean (SD)	0.80 (1.16)	0.72 (0.84)	0.099	0.70 (1.12)	0.69 (0.78)	0.817	0.90 (1.19)	0.78 (0.92)	0.077
Pre-dilatation, n (%)	814 (94.2)	1096 (89.9)	0.001	421 (93.1)	623 (86.4)	< 0.001	393 (95.4)	473 (95.0)	0.896
Pre-dilation balloon diameter, median [IQR]	2.50 [2.00. 3.00]	2.50 [2.00. 3.00]	0.531	2.00 [2.00. 2.50]	2.00 [2.00. 2.50]	0.438	3.00 [2.50. 3.50]	3.00 [2.50. 3.50]	0.127
SCB length, mean (SD)	22.38 (7.63)	22.03 (7.43)	0.297	23.42 (8.12)	22.39 (8.01)	0.033	21.24 (6.88)	21.52 (6.47)	0.534
SCB diameter, mean (SD)	2.63 (0.56)	2.65 (0.55)	0.523	2.28 (0.37)	2.35 (0.39)	0.003	3.02 (0.48)	3.07 (0.47)	0.075
SCB pressure of inflation, mean (SD)	9.87 (4.14)	9.92 (4.55)	0.819	8.89 (3.31)	9.23 (4.59)	0.172	10.95 (4.66)	10.91 (4.31)	0.892
SCB Inflation time, mean (SD)	57.83 (14.78)	57.74 (22.28)	0.915	58.88 (15.45)	58.19 (26.84)	0.618	56.67 (13.93)	57.08 (13.18)	0.651
Procedural complication, n (%)	12 (1.4)	20 (1.6)	0.780	7 (1.5)	12 (1.7)	0.999	5 (1.2)	8 (1.6)	0.829
Bailout stenting	60 (6.9)	101 (8.3)	0.258	37 (8.2)	66 (9.2)	0.569	23 (5.6)	35 (7.0)	0.323
Dissection (%)	31 (3.6)	42 (3.4)	0.957	26 (5.8)	37 (5.1)	0.745	5 (1.2)	5 (1.0)	0.999
Angiographic success (%)	846 (97.9)	1186 (97.3)	0.445	437 (96.7)	698 (96.8)	0.999	409 (99.3)	488 (98.0)	0.181

DM: diabetes mellitus; ISR: in-stent restenosis; PCI: percutaneous coronary intervention; SCB: sirolimus-coated balloon

**Fig. 1:**
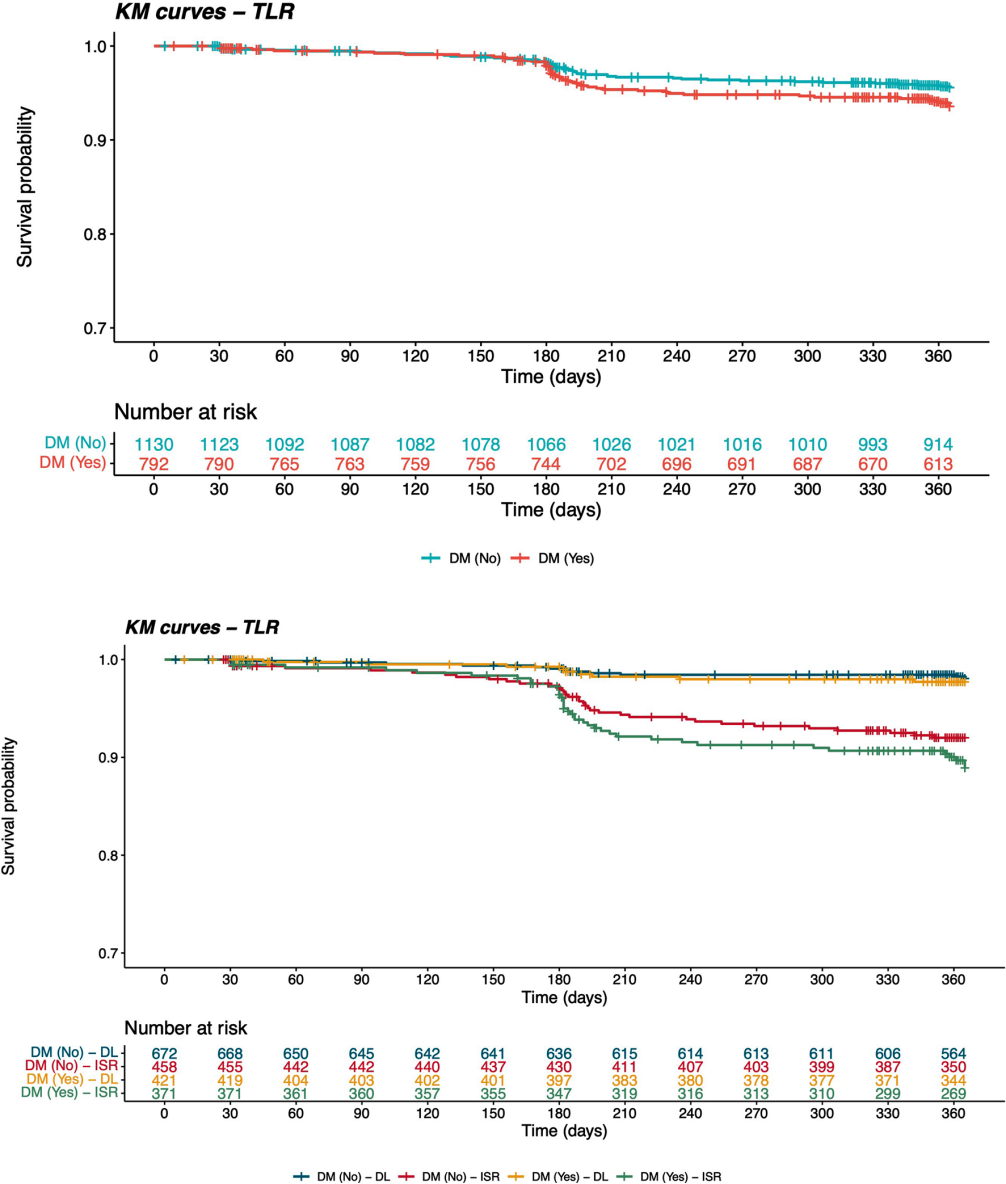
Cumulative incidence of target lesion revascularization at 12-month among patients stratified according diabetic status (panel A) and type of lesions treated (de novo lesions or ISR; panel B). DM: diabetes mellitus; DL: de-novo lesions; ISR: in-stent restenosis

Clinical outcomes at 12-month follow-up are reported in Table 3. At 1-year, diabetic patients suffered from a significantly higher rate of spontaneous MI (3.4% vs. 1.5% HR 2.15 95%CI 1.09–4.25). Diabetic patients had a higher albeit not statistically significant risk of the occurrence of the primary endpoint TLR (6.5% vs. 4.7% per-lesion analysis; HR 1.38, 95%CI 0.91–2.08; Fig. 1A), target vessel revascularization (6.0% vs. 5.0% per-patient analysis; HR 1.15 95%CI 0.73–1.81), all-cause death (3.8% vs. 2.6%, HR 1.81, 95%CI 0.95–3.46), and MACE (12.2% vs. 8.9%, HR 1.26 95%CI 0.92–1.74). No statistically significant differences in terms of BARC bleedings were evident among diabetic and non-diabetic patients (1.0% vs. 0.3%; HR 2.65 95%CI 0.75–9.31); of note, 34% of patients were on DAPT at 1-year follow-up with no significant differences between the two groups.

**Table 3:** Clinical outcomes at 12-month follow-up stratified according to diabetic status and type of lesion (de-novo lesions vs. ISR)

	Overall population (*N* = 2,083)			Patents with de-novo lesions (*N* = 1,173)			Patients with ISR (*N* = 910)		
	Patients with DM (*N* = 864)	Patients without DM (*N* = 1,219)	HR (95% CI)	Patients with DM (*N* = 452)	Patients without DM (*N* = 721)	HR (95% CI)	Patients with DM (*N* = 412)	Patients without DM (*N* = 498)	HR (95% CI)
Death, n (%)	33 (3.8)	32 (2.6)	1.81 (0.95–3.46)	10 (2.2)	14 (1.9)	1.80 (0.58–5.64)	23 (5.6)	18 (3.6)	1.74 (0.79–3.81)
MI, n (%)	29 (3.4)	18 (1.5)	2.15 (1.09–4.25)	8 (1.8)	3 (0.4)	2.44 (0.57–10.46)	21 (5.1)	15 (3.0)	1.90 (0.88–4.09)
Bleeding, n (%)	9 (1.0)	4 (0.3)	2.65 (0.75–9.31)	3 (0.7)	3 (0.4)	1.49 (0.29–7.60)	6 (1.5)	1 (0.2)	7.26 (0.71–74.20)
MACE, n (%)	105 (12.2)	109 (8.9)	1.26 (0.92–1.74)	27 (6.0)	40 (5.5)	1.05 (0.59–1.85)	78 (18.9)	69 (13.9)	1.39 (0.94–2.04)
TLR, n (%), per-patient	52 (6.0)	61 (5.0)	1.15 (0.73–1.81)	9 (2.0)	13 (1.8)	1.00 (0.39–2.59)	43 (10.4)	48 (9.6)	1.13 (0.67–1.90)
TLR, n/lesion number (%), per lesion	63/968 (6.5)	64/1371 (4.7)	1.38 (0.91–2.08)	11/486 (2.3)	13/798 (1.6)	1.35 (0.56–3.26)	52/482 (10.8)	51/573 (8.9)	1.36 (0.85–2.16)

DM: diabetes mellitus; ISR: in-stent restenosis; MI: myocardial infarction; MACE: major adverse cardiovascular event; TLR: target lesion revascularization; Cox model HR (95% CI) are shown; adjusted for patient age, hypercolesterolemia, hypertension, MVD and predilatation

A total of 1,173 (56.3%) patients underwent PCI with SCB for de-novo coronary lesions, of these 452 (38.5%) had diabetes. At 12-month follow-up, as compared to non-diabetic, diabetic patients had similar rates of death, TLR, bleeding and MACE. Spontaneous MI occurred numerically more frequently in diabetic patients (1.8% vs. 0.4%, HR 2.44, 95%CI 0.57–10.46). The overall incidence of TLR was lower in de-novo coronary lesions group, without any significant difference between diabetic and non-diabetic patients (2.3% vs. 1.6%, per-lesion analysis, HR 1.35, 95%CI 0.56–3.26) (Table 3; Fig. 1B).(See Central illustration).

**Central illustration:**
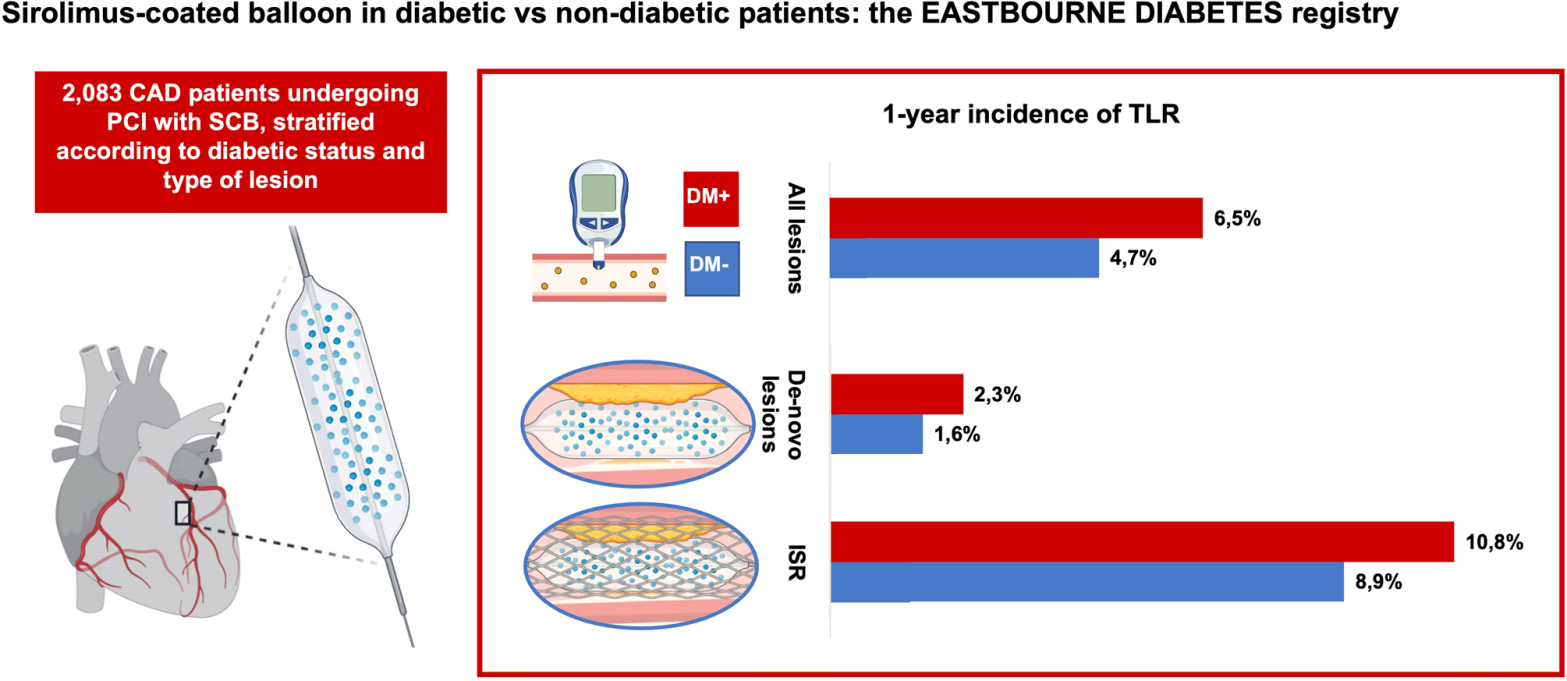
One-year incidence of per-lesion TLR among patients treated with sirolimus-coated balloon, stratified according to diabetic status and type of lesion. TLR: target lesion revascularization

Among 910 (43.7%) patients undergoing PCI for ISR, 412 (45.3%) had diabetes. At 12-month follow-up, diabetic patients had similar rates of death, MI, TLR, and MACE. Risk of bleeding was numerically higher in the diabetic group (1.5% vs. 0.2%, HR 7.26, 95%CI 0.71–74.20). The overall incidence of TLR was higher in the ISR group, without any significant difference between diabetic and non-diabetic patients (10.8% vs. 8.9%, per-lesion analysis HR 1.36, 95%CI 0.85–2.16) (Table 3; Fig. 1B).

## Discussion

This pre-specified sub-group analysis of the EASTBOURNE registry aimed to evaluate the performance of the novel sirolimus-coated balloon MagicTouch in diabetic patients. The main findings of our analysis can be synthesized as follow:

- Diabetic patients treated with SCB did not have a significant increase in TLR when compared to non-diabetic patients.
- The risk of MI was significantly higher in patients with diabetes compared to non-diabetic patients up to 12 months; the incidence of death, TLR and MACE was only numerically higher.
- When stratifying the results for the type of treated lesion (de novo vs. ISR), a good performance of the study device in terms of MACE and TLR was evident.

The rationale for our prespecified analysis lies in the evidence that diabetes still represents a major cardiovascular risk factor despite the increased awareness of this pathology and the wider therapeutic armamentarium available nowadays. The incidence of diabetes has increased worldwide and its prevalence in CAD patients undergoing percutaneous coronary intervention (PCI) has been reported to be as high as 20–30%, with an increasing trend [14, 15]. 

Diabetic patients still experience significantly higher all-cause mortality rates than subjects without diabetes after adjustment for other risk factors and have a 2 to 4-fold increased risk of both coronary and peripheral artery disease [16–18]. 

In the setting of PCI with stent, there is solid evidence that diabetic patients have an accelerated rate of late lumen loss and that diabetes mellitus represents an independent predictor of recurrent restenosis [19]. From a clinical perspective, diabetic patients exhibit poor outcomes after stent angioplasty, with higher rates of stent thrombosis, myocardial infarction, and death irrespective of the DES type [20–22]. Explanations for such findings may also be found in the typical pattern of diabetic atherosclerosis, including more complex, diffuse, and long lesions affecting especially smaller caliber vessels with reduced coronary vasodilator reserve, as a consequence of higher degree of vascular inflammation and endothelial dysfunction [23]. The introduction of newer generation DES has mitigated such diabetes-associated issues but long-term follow-up studies demonstrated that the phenomenon cannot be eliminated [15, 24]. Ten-year clinical outcomes of the prespecified sub-groups of patients with and without diabetes mellitus in the ISAR-TEST 5 trial (comparing new generation DES in a randomized fashion) showed higher rate of events in diabetic patients and such events continue to accrue over time [24]. In a pre-specified sub-group analysis of the GLOBAL LEADERS trial, the risk of all-cause death, cardiac death, patient-oriented composite endpoint, ischemic stroke, any MI, and any revascularization were significantly higher in non-insulin treated diabetic patients than non-diabetics; of interest, the risk of adverse events was even higher in insulin-treated diabetics [25]. As a matter of fact, such findings are also substantiated by studies favoring CABG over PCI when treating diabetic patients with multivessel coronary artery disease [26]. In this scenario, the introduction of DCB for the treatment of coronary lesions has been welcomed since many years, following the concept that a “leave nothing behind” strategy might improve the outcome of patients undergoing PCI, especially in diabetic patients usually showing diffuse disease located in smaller vessels and a more reactive inflammatory response following coronary stents implantation. By the absence of a permanent vascular metallic implant, the risk of late or very late stent thrombosis is prevented and the need for ìDAPT could be reduced. Moreover, by allowing the even distribution of the antiproliferative drug along the vessel wall, some paclitaxel-eluting DCB have shown to promote positive remodeling; however, the main drawback of this technology is represented by the risk of suboptimal results due to persistent residual stenosis, acute vessel recoil and dissections [27]. A growing body of evidence is testing the efficacy of DCB which demonstrated to be non-inferior to DES also in complex lesions like those located in small or mid-sized coronary vessels as reported in the Long-term Efficacy and Safety of Drug-Coated Balloons versus Drug-Eluting Stents for Small Coronary Artery Disease (BASKET-SMALL 2) trial [8]. Interestingly, a recent sub-analysis from the same group of investigators evaluated the impact of diabetes mellitus on long-term clinical outcomes in this setting and found that the rates of MACE were similar in both diabetic and nondiabetic patients; however, in diabetic patients, the need for TLR was significantly lower with DCB versus DES [6]. Large registries have been conceived to test the safety and the efficacy of both SCB and paclitaxel-coated balloons (PCB) in the coronary setting [28]. Of note, because the interaction among antiproliferative doses and release kinetics of the drug is important, a “class effect” cannot be claimed for DCB [7]. A first indirect comparison between the MagicTouch SCB and PCB could not find significant differences in terms of safety and efficacy; at multivariable analysis, diabetes remained the only independent predictor of MACE [29]. The SCB used in our study is the first sirolimus-based balloon marketed in EU and has beenXXXrevioussly tested in small registries also in complex scenarios like ACS patients [30–33]. Two RCTs have been conceived to test the MagicTouch balloon [34, 35]. The TRANSFORM I randomized 120 patients to PCB Sequent Please (Bbraun, Germany) and MagicTouch after lesion assessment with optical coherence tomography, evaluating net lumen gain at 6 months angiographic follow up [34]. The TRANSFORM II is randomizing patients to everolimus-eluting stents and MagicTouch and will test if this device will be non-inferior to DES in terms of TLF at 1 year (primary endpoint) and subsequently through the 5-year follow up [35]. Of note, the 12-month follow-up of the EASTBOURNE registry, the largest on DCB so far, recently demonstrated good immediate performance and an adequate and encouraging safety profile of the MagicTouch balloon, used to treat a wide spectrum of coronary lesions in an all comers setting [13]. Diabetes is widely represented in the EASTBOURNE registry population with 41.5% of diabetic patients. Our findings corroborate the concept that the MagicTouch SCB is a safe and effective device for the treatment of coronary lesions also in the more complex scenario of diabetic patients, with a TLR rate as low as 6.5%, not significantly different from non-diabetics. The real-world nature of our registry is confirmed by the complexity of the patients treated, with the diabetic sub-group showing increased risk of hypercholesterolemia, hypertension, multivessel disease, calcific lesions and ISR. As expected, due to the detrimental systemic effects of diabetes, a significantly increased risk of MI and a numerically higher risk of all-cause death and MACE were reported, as also DES-based studies historically report. In our study, included patients were relatively young; however, their age and characteristics aligned with those observed in other similar studies within this field. However, since elderly patients are often under-treated, being considered a population at risk for antithrombotic therapy and treatment with stents, especially for small vessels disease, future studies might explore the benefit of DCB use in this subgroup. Of note, after stratification for the type of treated lesion (de novo vs. ISR), our study still shows a good performance of the study device in terms of MACE and TLR. However, non-univocal data exist, especially focusing on the comparison between DCB and DES in diabetics. A recent meta-analysis including 378 patients from three studies comparing DCB vs. DES after PCI of de-novo coronary lesions in diabetic patients, found a similar risk of MACE, TLR, binary restenosis and late lumen loss at 17.3 ± 11.3 months follow-up [36]. However, the small number of studies mainly including old-generation paclitaxel-eluting stents might limit the impact of such findings.

## Limitations

Some limitations inherent to the main EASTBOURNE prospective registry may also apply to the present analysis. First, it was a single-arm open-label registry and the decision to use SCB was left to the operator’s discretion; thus, the lack of randomization might have affected the results because of the presence of unmeasured confounding factors. Second, among patients with de-novo coronary lesions, SCB use was mainly restricted to small caliber vessels and results may not be generalized to larger diameter arteries. Third, nearly 90% of the lesions received a preparation before SCB but the implementation of modern lesion preparation devices (e.g., noncompliant or scoring balloons, intravascular lithotripsy) was low and up to 20% of screened patients did not undergo SCB PCI due to flow-limiting dissection or residual stenosis > 50%. Finally, the lack of information on the duration of diabetes, glycemic control and on diabetes medications represent specific limitations of the present study. For all these reasons, our analyses should be considered exploratory, and the results should be interpreted with caution as hypothesis-generating.

## Conclusions

The EASTBOURNE DIABETES study supports the use of SCB for the treatment of both de novo and ISR lesions in the complex setting of diabetic patients, adding important data to field and paving the way for larger dedicated trials.

## Data Availability

All data generated or analyzed during this study are included in this published article.

## Abbreviations

PCI: Percutaneous Coronary Intervention
CAD: Coronary Artery Disease
SCB: Sirolimus-coated balloon
TLR: Target Lesion Revascularization
MACE: Major Adverse Clinical Events
MI: Myocardial Infarction
ACS: Acute Coronary Syndrome
DES: Drug-eluting Stent
DCB: Drug-coated Balloon
ISR: In-stent restenosis
BARC: Bleeding Academic Research Consortium
